# Hirsutine, an Emerging Natural Product with Promising Therapeutic Benefits: A Systematic Review

**DOI:** 10.3390/molecules28166141

**Published:** 2023-08-19

**Authors:** Md. Shimul Bhuia, Polrat Wilairatana, Jannatul Ferdous, Raihan Chowdhury, Mehedi Hasan Bappi, Md Anisur Rahman, Mohammad S. Mubarak, Muhammad Torequl Islam

**Affiliations:** 1 Department of Pharmacy, Bangabandhu Sheikh Mujibur Rahman Science and Technology University, Gopalganj 8100, Bangladesh; shimulbhuia.pharm@gmail.com (M.S.B.); raihanpharmacy049@gmail.com (R.C.); mehedibappi22@gmail.com (M.H.B.); 2 Department of Clinical Tropical Medicine, Faculty of Tropical Medicine, Mahidol University, Bangkok 10400, Thailand; 3 Department of Biotechnology and Genetic Engineering, Bangabandhu Sheikh Mujibur Rahman, Science and Technology University, Gopalganj 8100, Bangladesh; jannat16bge093@gmail.com; 4 Department of Pharmacy, Islamic University, Kushtia 7003, Bangladesh; anisurrahmaniupharm@gmail.com; 5 Department of Chemistry, The University of Jordan, Amman 11942, Jordan; mmubarak@ju.edu.jo

**Keywords:** hirsutine, pharmacological activities, cancer, diabetes, anti-hypertensive effect, pharmacokinetics

## Abstract

Fruits and vegetables are used not only for nutritional purposes but also as therapeutics to treat various diseases and ailments. These food items are prominent sources of phytochemicals that exhibit chemopreventive and therapeutic effects against several diseases. Hirsutine (HSN) is a naturally occurring indole alkaloid found in various Uncaria species and has a multitude of therapeutic benefits. It is found in foodstuffs such as fish, seafood, meat, poultry, dairy, and some grain products among other things. In addition, it is present in fruits and vegetables including corn, cauliflower, mushrooms, potatoes, bamboo shoots, bananas, cantaloupe, and citrus fruits. The primary emphasis of this study is to summarize the pharmacological activities and the underlying mechanisms of HSN against different diseases, as well as the biopharmaceutical features. For this, data were collected (up to date as of 1 July 2023) from various reliable and authentic literature by searching different academic search engines, including PubMed, Springer Link, Scopus, Wiley Online, Web of Science, ScienceDirect, and Google Scholar. Findings indicated that HSN exerts several effects in various preclinical and pharmacological experimental systems. It exhibits anti-inflammatory, antiviral, anti-diabetic, and antioxidant activities with beneficial effects in neurological and cardiovascular diseases. Our findings also indicate that HSN exerts promising anticancer potentials via several molecular mechanisms, including apoptotic cell death, induction of oxidative stress, cytotoxic effect, anti-proliferative effect, genotoxic effect, and inhibition of cancer cell migration and invasion against various cancers such as lung, breast, and antitumor effects in human T-cell leukemia. Taken all together, findings from this study show that HSN can be a promising therapeutic agent to treat various diseases including cancer.

## 1. Introduction

The term natural products (NPs) refers to substances that originate from natural sources such as plants, animals, and microorganisms. Many of these compounds possess important biological properties [1]. Research findings showed that NPs have provided valuable starting points for the development of numerous highly successful medications that are presently used to treat various illnesses in humans [2]. For centuries, NPs have been utilized as traditional remedies, medicines, potions, and oils without any understanding of the bioactive compounds they contain. People have relied solely on the results of centuries of experimentation to determine their effectiveness [3].

Compared to conventional synthetic molecules, NPs have distinct characteristics that present both benefits and obstacles during the drug discovery process [4]. NPs represent a significant reservoir of orally available medications [5]. In contrast, synthetic drugs are prepared in the laboratory using various methods that have the maximum potential to be toxic or cause side effects in addition to their therapeutic benefits. While herbal medicines may not be as potent as synthetic drugs in certain cases, they are generally considered to be less toxic or cause fewer side effects when compared to synthetic medications [6,7]. In this regard, herbal medications are frequently used in holistic treatments intended to cure a wide variety of diseases as they fulfill the primary criteria for being reliable therapeutics, such as being efficacious and non-toxic. Therefore, numerous chemists are shifting from synthetic chemistry to natural product research to further investigate nature’s wonders [8,9].

In the history of drug development from natural sources, Alexander Fleming made the most significant contribution to the field of natural substances at the start of the 20th century through his discovery of penicillin in 1928. Penicillin, derived from *Penicillium chrysogenum*, is a molecule with antibiotic properties and forms the basis of modern anti-lactam antibiotics. The discovery of penicillin led to a concentration of scientific research on isolating NPs from microbial and other sources [10,11]. During this period, various modern pharmaceutical industries focused on developing more antibiotics along with other drugs such as streptomycin, gentamicin, and tetracycline from natural sources on the basis of microbial fermentation technologies [1,12].

Indole alkaloids, which are abundant and a major source of pharmacologically active substances, have had a considerable impact on the preparation of novel anti-cancer medications such as drugs like vincristine and vinblastine [13]. Indole alkaloids are found in various plants such as *Catharanthus roseus* and *Alstonia scholaris*, which exhibited a number of therapeutic properties in addition to their anticancer properties in various preclinical tests. An additional alkaloid, ajmalicine, exhibits anti-arrhythmia and antihypertensive properties, while catharanthine and vindoline have demonstrated diuretic effects, antibacterial activities, and antidiabetic properties derived from plants [14]. Another alkaloid, dragmacidin D, which is an indole-containing alkaloid, can inhibit the development of certain gram-negative and gram-positive microorganisms such as *Bacillus subtilis* and *Escherichia coli*. Furthermore, it has been demonstrated that dragmacidin D can suppress the growth of several opportunistic yeasts like *Candida aeruginosa*, *Candida albicans*, and *Candida neoformans* [15]. Makaluvamine G possesses a moderate ability to inhibit topoisomerase I and can also suppress the activity of DNA and proteins [15].

**Figure 1:**
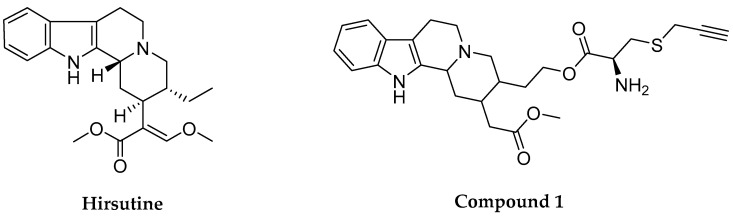
Chemical structures of hirsutine and its derivative (Compound **1**).

Hirsutine (HSN) (Figure 1) is the primary indole alkaloid found in Uncaria species, mainly in *Uncaria rhynchophylla*. These plants of the genera are used in local Chinese herbal medicine to treat a variety of symptoms involved with hypertension and cerebrovascular disorders, including spasmolytic, analgesic, and sedative treatments [16]. Published research showed that HSN exhibits a depressant effect on the central nervous system (CNS) in mice, a weak antispasmodic effect on the intestine of mice, and a lowering effect on blood pressure in rats [17]. Pharmacological studies have shown that HSN may have therapeutic benefits in treating certain conditions as it exerts anti-inflammatory (preeclampsia) [18], antiviral (dengue fever and dengue hemorrhagic fever) [19], anticancer activity (breast and lung cancers) [20,21,22,23,24], antitumor [25], anti-diabetic [26], antihypertensive, negative chronotropic, and antiarrhythmic activity [27]. It can also be used to treat and manage myocardial infarction, ischemia-reperfusion (I/R) trauma [28], brain inflammation [29], cerebral ischemia [30], cardiovascular diseases (cardiomyocytes) [31], anti-hypertension [14,32], hypotensive and vasodilatory effects [33], lung cancer [34], thrombolytic effects (thrombocytopenia) [35], and neurogenerative diseases and disorders such as neuronal death [36]. Based on the previous discussion, the aim of the present work was to summarize the literature dealing with the pharmacological effects of HSN and present an overview of the recent developments of its use in the prevention and treatment of different diseases. We hope findings from this can help guide future research and aid in the development of new therapeutic strategies. 

## 2. Results

### 2.1. Botanical Sources of Hirsutine

Plants are a significant source of medication and are crucial to global health. Medicinal plants play a critical role in protecting from diseases and are integrated into preventive programs, not only for the treatment of illness but as a possible material for preserving good well-being and conditions [37]. Numerous compounds found in plants have been utilized in medicine to aid in the development of novel drugs [38,39]. The phytochemical HSN, which is a type of indole alkaloid, has been obtained from different species of the Uncaria genus. This plant belongs to the Rubiaceae family [31]. In this respect, various species such as *Uncaria rhynchophylla* (Miquel), *U. lancifolia*, *U. hirsuta*, *U. scandens*, *U. homomalla*, *U. sessilifructus*, *U. laevigata*, *U. macrophylla*, *U. yunnanensis*, *U. lanosa*, and *U. rhynchophylloides*, are rich sources of HSN [25,33,40,41,42,43,44]. The species *U. rhynchophylla* has gained attention for its various biological traits such as cardioprotective, antihypertensive, and antiarrhythmic effects [14]. Throughout history, the bark of *U. rhynchophylla* has been used as a remedy for various ailments including convulsions, bleeding, hypertension, autoimmune disorders, and cancer [25]. Several other studies suggested that the plant *U. rhynchophylla* (Miquel) exhibits various pharmaceutical effects such as antihypertensive, anti-inflammatory activity, sedative, and antiarrhythmic actions due to the presence of HSN [18,32]. The other sources of HSN are *U. sinensis* [36], *U. tomentosa* [45], and *Mitragyna hirsute* (https://pubchem.ncbi.nlm.nih.gov/taxonomy/371154, accessed on 30 April 2023). Listed in Table 1 are the botanical sources and the plant portion where HSN is found in significant quantities.

### 2.2. Physicochemical and Biopharmaceutical Profiles 

The role of pharmacokinetics (PKs) in drug discovery is to support the optimization of lead compounds’ absorption, distribution, metabolism, and excretion (ADME) properties to develop a clinical candidate that has a concentration-time profile in the body that is sufficient for the sought efficacy and safety profile [46,47]. Bioavailability is referred to as the extent and rate of absorption and availability of the active drug constituent or active moiety from the drug product at the site of drug action [48,49]. The main reasons some drug candidates fail in clinical trials are due to undesirable PK features and unacceptable toxicity [50]. Therefore, it is important to focus on optimizing and characterizing human ADME features and understanding the pharmacokinetic-pharmacodynamic relationship to estimate a clinically relevant dose [51].

HSN (C_22_H_28_N_2_O_3_) is a white crystalline powder with a density of 1.20 ± 0.1 g/cm^3^ and a molecular mass of 368.5 g/mol. The melting point of the compound is 101 °C, while the boiling point is 531.7 ± 50.0 °C (Predicted). Since HSN is readily soluble in methanol, acid water, chloroform, and lipids, its concentrations rapidly decline as the tissues metabolized the hormone [52], (https://pubchem.ncbi.nlm.nih.gov/compound/3037884, accessed on 4 May 2023; https://www.chembk.com/en/chem/Hirsutine, accessed on 10 July 2023). Research findings showed that the bioavailability of HSN is 4.4% and clearance (CL) is remarkably lower than that of oral administration. In addition, findings indicated that HSN has poor absorption. For oral administration, the volume of distribution (VD), mean residence time (MRT), the highest concentration of a drug in the blood, cerebrospinal fluid, or target organ after a dose is given (C_max_), and CL of HSN were 70.8 ± 17.8 ng/mL, 3.6 ± 0.6 h, and 21.9 ± 6.6 ng/mL, respectively [52]. In humans, the half-life of HSN in plasma is 3.4 h, and the time it takes to reach its maximum concentration (t_max_) is 0.50 to 0.83 h [53]. HSN is distributed to the brain, liver, kidney, spleen, heart, and lungs. The liver and kidney tissues attain the highest distribution levels, followed by the lung and spleen. HSN exhibits a modest concentration in brain tissue but was widely distributed, indicating that it might cross the blood-brain barrier (BBB) [52]. Another investigation by Gai et al. (2020) showed that HSN has the capability to cross BBB. These researchers found that HSS inhibits P-glycoprotein mRNA expression in MCF-7/ADR cells [44]. HSN is mostly metabolized in liver tissues before being eliminated in renal tissues, which may be associated with high levels of blood flow and tissue oxygenation in these organs. Furthermore, HSN is metabolized by cytochrome P450s (CYPs) in rat liver microsomes [16]. In rats, HSN undergoes glucuronidation to produce 11-hydroxy metabolites, which are primarily eliminated in bile rather than urine [16].

In our in silico ADME prediction, HSN exhibited better ADME properties to be considered a drug candidate. HSN followed Lipinski’s rule of 5, such as H-bond (HB) acceptors (4) and HB donors (1), as well as molar refractivity (MR) 110.39 and TPSA (54.56 Å^2^) where all the parameters are within the limit (HB acceptors ≤ 10, HB donors ≤ 5, MR ≤ 140, TPSA ≤ 140 Å^2^) indicating better physiochemical properties for better ADME as there is no violation of Lipinski’s rule of 5 [49] (http://www.swissadme.ch/index.php, accessed on 10 August 2023). ADME prediction also demonstrated that HSN is moderately water soluble, highly absorbable through GIT, capable of permitting BBB, and inhibits P-gp, which is almost the same as reported by different in vivo studies [44,52] (http://www.swissadme.ch/index.php, accessed on 10 August 2023). Different ADME parameters of HSN predicted by SwissADME are shown in Table 2.

### 2.3. Pharmacological Profile of Hirsutine

#### 2.3.1. Neurobiological Effects

##### Prevention of Neuroinflammation and Neurotoxicity

Inflammation is a natural immunological reaction that can be provoked by infectious agents, toxic compounds, and damaged cells, among others. These factors can trigger chronic and/or acute inflammatory reactions in the pancreas, heart, liver, lung, kidney, urinary tract, brain, gastrointestinal tract, respiratory tract, and reproductive organs, which may cause tissue injury or disease [54,55]. Immune activation within the CNS is a defining characteristic of ischemia, immune-mediated disorders, neurodegenerative diseases, infections, and trauma, and can frequently cause neuronal injury [56]. Immune and inflammatory responses in CNS are principally mediated by microglia [57]. Activated microglia also contribute to neuronal damage via the secretion of proinflammatory and cytotoxic factors, such as cytokines, reactive oxygen species (ROS), and nitrogen oxides (NO) [58,59]. Microglia can be activated by lipopolysaccharide (LPS), which is a strong immunogenic particle released from gram-negative bacteria and induces inflammation in various experimental animals and cell lines [60]. Mice with SIRS induced by LPS revealed a systemic and local inflammatory response through the discharge of different inflammatory enzymes and cytokines [61].

Published research demonstrated that HSN blocks LPS-related hippocampal cell death and the generation of NO, PGE2, and IL-1β. It also revealed that HSN effectively hinders LPS-mediated NO secretion from cultured rat brain microglia and results in diminished production of PGE2 and intracellular ROS generation, resulting in diminishing neurotoxicity (Table 3) [29]. Another study by Wu et al. (2019) reported that treatment with *Uncaria rhynchophylla* alkaloid extract (where HSN presents as an active phytochemical) in LPS-mediated preeclampsia rats inhibited the level of proinflammatory cytokines (IL-6, IL-1β, TNF-α, and IFN-γ), resulting in therapeutic benefits in the complication of pregnant rats by reducing inflammation [18].

##### Prevention of Neuronal Cell Death

Glutamate is the primary stimulating neurotransmitter in the CNS and is essential in learning, memory development, and metabolism in the brain [62,63]. However, excessive glutamate causes the death of central neurons in various pathological situations such as ischemic-hypoxic injury, epilepsy, and neurodegenerative illness. Various studies reported that ischemia promotes excessive release of glutamate, which stimulates the inotropic glutamate receptors, and overstimulated receptors cause Ca^2+^ influx [64,65,66]. Ca^2+^ influx triggers cell death by raising the activity of Ca^2+^-dependent enzymes, including phospholipase C and protein kinase C, which degrade cytoskeletal proteins [67,68]. It is also evident that there is a link between the increase in ROS generation and the inflow of Ca^2+^ into cells [69,70]. In addition, research findings indicated that HSN (3 × 10 ^−4^–10 ^−3^ M) presents as a bioactive compound in alkaloids extracted from *Uncaria sinensis* is capable of diminishing glutamate-mediated death of neuronal cells in cultured cerebellar granule cells of rats by suppressing Ca^2+^ influx [36]. 

#### 2.3.2. Cardioprotective Activity

Acute myocardial infarction (AMI) is one of the major causes of disability and mortality in the world [71]. It causes irreparable damage to the cardiac muscle due to an insufficient supply of oxygen (hypoxia) and induction of oxidative stress, which is caused by ischemic reperfusion (I/R) injury [72]. The intracellular changes that occur during I/R, include the accumulation of H^+^ and Ca^2+^ and the disruption of the mitochondrial membrane potential, resulting in the production of free radicals or ROS. Accumulation of ROS and consequent activation of pro-inflammatory pathways play a key role in I/R injury. Therefore, oxygen-derived free radicals are a crucial mediator of I/R injury, leading to various forms of oxygen species [73,74], resulting in the apoptosis of cardiac cells [73,75]. I/R injury can be treated by phytochemicals, which can inhibit oxidative stress and has anti-apoptotic and anti-inflammatory effects [76]. 

An in vivo experiment by Jiang et al. (2023) demonstrated that HSN plays a protective role in inhibiting apoptosis in I/R injury. Pretreatment with HSN (5, 10, and 20 mg/kg) in I/R injured AMI rats revealed reductions in myocardial infarct size, mitochondrial function, and histological injury, and inhibited cardiac cell apoptosis by hindering the AKT/ASK-1/p38 MAPK pathway. Moreover, the results of the study showed that HSN enhances cardiac function and diminishes tissue lactate dehydrogenase (LDH) and ROS content [28]. Another study by Wu et al. (2011) showed that HSN exerts cardioprotective effects due to its antioxidant and anti-apoptotic effects. Additionally, HSN (0.1, 1, and 10 μΜ) prevented hypoxia-induced myocyte cell death by regulating proapoptotic signaling cascades associated with Bcl-2 family proteins and caspases which inhibit the destruction of hypoxia-induced myocytes. Furthermore, HSN suppressed ROS generation by enhancing the activity of antioxidant enzymes and inhibited lipid peroxidation [31], resulting in the prevention of AMI as the destruction of myocytes is minimized.

#### 2.3.3. Antiviral Activity 

The lack of effective treatments for viral diseases is a significant health concern in the current era [77]. Among the different viruses, dengue and flu (influenza) cause major health concerns. Dengue is a viral infection that, in extreme circumstances, can be fatal [78]. The incidence of dengue is on the rise, and the disease is a significant public health concern in tropical regions. Each rainy season is accompanied by a surge of dengue epidemics, where thousands could be affected [79,80]. On the other hand, in humans, influenza viruses produce two distinct types of respiratory illness: the seasonal variety and the pandemic variety [81], especially in infants. However, it is a matter of main concern that resistance to antiviral drugs leads to the development of new drugs. 

**Figure 2:**
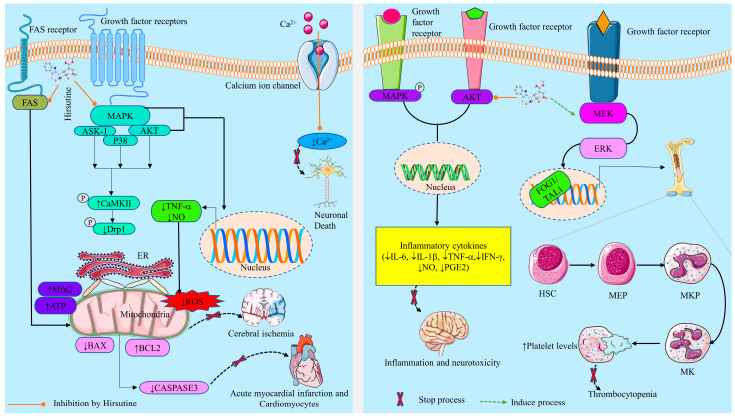
The underlying mechanisms of different pharmacological effects of hirsutine. ↓: Decrease/inhibition/downregulation; ↑: Increase/upregulation/stimulation; HSC: Hematopoietic Stem Cell; MEP: Megakaryocyte-Erythroid Progenitor; MKP: Mitogen-Activated Protein Kinase Phosphatase; NO: Nitric Oxide; PGE2: Prostaglandin E2; IL-6: Interleukin-6; IL-1β: Interleukin-1 beta; TNF-α: Tumor Necrosis Factor-alpha; IFN-γ: Interferon-gamma; ROS: Reactive Oxygen Species.

HSN (10 µM) demonstrated an antiviral effect against all the dengue virus (DENV) serotypes ((DENV-1 (02-20 strain), DENV-2 (16681 strain), DENV-3 (09-59 strain), and DENV-4 (09-48 strain)) by preventing the viral particle assembly, budding, or release phases in the DENV lifecycle, but not the viral translation and replication steps. Using a subgenomic replicon system, it was determined that HSN does not hinder viral genome RNA replication. Additionally, studies suggested that the antiviral activity of HSN may be connected to calcium homeostasis [16]. The antiviral activity of HSN was also evident in a study by Takayama et al. (1997); the results of this study indicated that HSN effectively suppresses the replication of the strains of influenza A (subtype H3N2) with the EC_50_ of 0.4–0.57 µg/mL [82]. Depicted in Figure 2 are the underlying mechanisms of different pharmacological effects of HSN.

#### 2.3.4. Anticancer Activity of Hirsutine: Underlying Mechanisms

##### Induction of Oxidative Stress

An elevated degree of oxidative stress is regarded as a novel target of anticancer therapy. This can be triggered by raising exogenous ROS or blocking the endogenous antioxidant defense system [83,84]. ROS levels can rise as a result of chemotherapy medications [85], which may amplify the cytotoxic effects of the chemotherapeutic agents. ROS are highly reactive molecules that can lead to oxidative damage to cellular components such as proteins, DNA, and lipids [86]. In addition, the elevation in ROS levels can lead to the activation of various signaling pathways, including the p53 pathway, which can induce apoptosis in cancer cells. A recent study found that HSN causes mitochondrial death by causing ATP depletion, the formation of ROS, the loss of mitochondrial membrane potential, and the discharge of cytochrome C (Cyt C) [34]. Additionally, to overcome HSN resistance, it may be helpful to disrupt the ataxia telangiectasia mutated (ATM) pathway, which results in ROS production and a p53-independent DNA damage response in breast cancer cells [21].

##### Cytotoxicity

Cytotoxicity is the ability of a substance, such as a chemical or drug, to kill or damage cells. Many chemotherapeutic drugs work by exerting cytotoxic effects on rapidly dividing cancer cells, thereby inhibiting their growth and causing them to die [84,87]. In this context, findings revealed that HSN exerts cytotoxic effects against different cell lines in multiple investigations, which raises the possibility of its development as an anticancer drug. According to a study by Meng et al. (2021), HCN showed cytotoxicity against Jurkat clone E6-1 tumor cells [25]. In a different study, it was discovered that HSN exhibits strong cytotoxicity against human breast cancer cell lines, including MCF-7, MDA-MB-231, MCF-10A, BT474, and MDA-MB-453 with concentrations ranging from 6.25−160 M (Table 3) [20,21,22,23,24].

##### Apoptotic Effect

Chemotherapy drugs can induce apoptosis in cancer cells as a mechanism of their cytotoxicity [88]. Apoptosis is a tightly regulated process of programmed cell death that occurs in response to different stimuli, including chemotherapy drugs [89]. Induction of apoptosis in cancer cells is crucial in cancer treatment, as it allows for the selective elimination of cancer cells without affecting normal ones [90]. The Bcl-2 family protein is involved in the regulation of apoptotic cell death and inhibition of apoptosis [91]. On the other hand, findings revealed that Bax exhibits a potent apoptosis-promoting capacity, resulting in alterations in the membrane potential and structure of mitochondria, and then initiates the caspase-independent apoptotic response via the mitochondrial pathway. Simultaneously, Cyt C is released directly or indirectly from the mitochondria to the cytoplasm to initiate the caspase-dependent apoptotic response via the mitochondrial pathway. Therefore, Bcl-2/Bax plays a crucial role in modulating caspase-dependent and caspase-independent apoptosis induced via the mitochondrial pathway [91,92]. In contrast, the PI3K/AKT signaling pathway is a vital intracellular signal transduction pathway that plays a crucial function in regulating apoptosis and survival [93]. Thus, targeting these pathways is one of the most reliable approaches to anticancer drug design and development.

According to a research report, HSN may cause the human breast cancer MDA-MB-231 cells to undergo programmed cell death by lowering the Bcl-2 to Bax ratio, opening the mitochondrial permeability transition pore (MPTP), secreting Cyt C from the mitochondria, and activating caspases 9 and 3 [22]. Another investigation also found that the cancer cell death mechanism depends on mitochondria. At doses of 10, 25, and 50 µM/L, HSN inhibited Jurkat Clone E6-1 cell death by up-regulating Bcl-2 levels as a preventive compensatory mechanism. The Bax/Bcl-2 ratio may change to influence the apoptotic activity of HSN because HSN therapy markedly diminished Bcl-2 expression and elevated Bax expression [25]. According to another study, HSN suppresses tumor development and promotes apoptosis in an A549 xenograft mouse model via the ROCK1/PTEN/PI3K/Akt/GSK3 signaling pathway [34]. Furthermore, HSN triggers cell apoptosis by mediating DNA damage and inhibiting breast cancer (MCF-7) cell lines [21]. Moreover, HSN causes apoptotic cell death by activating caspases. Additionally, HSN promoted a DNA damage response in MDA-MB-453 cells, as evidenced by an increase in γH2AX expression [22].

##### Inhibition of Cell Migration and Invasion

Inhibition of cell migration and invasion is an important therapeutic goal in cancer treatment, as the ability of cancer cells to spread and invade surrounding tissues is a primary factor in the progression of the disease [94,95]. While chemotherapy drugs primarily target rapidly dividing cells, some drugs may also possess properties that can inhibit cell migration and invasion [96]. In addition to promoting tumor cell proliferation, inhibiting apoptosis, and attracting angiogenesis, NF-κB activity induces epithelial-mesenchymal transition, thereby facilitating distant metastasis. Under specific conditions, NF-κB activation may also remodel local metabolism and depress the immune system to promote tumor growth. The NF-κB pathway is a prospective therapeutic target because inhibition of NF-κB in myeloid cells or tumor cells typically results in tumor regression [97,98].

**Figure 3:**
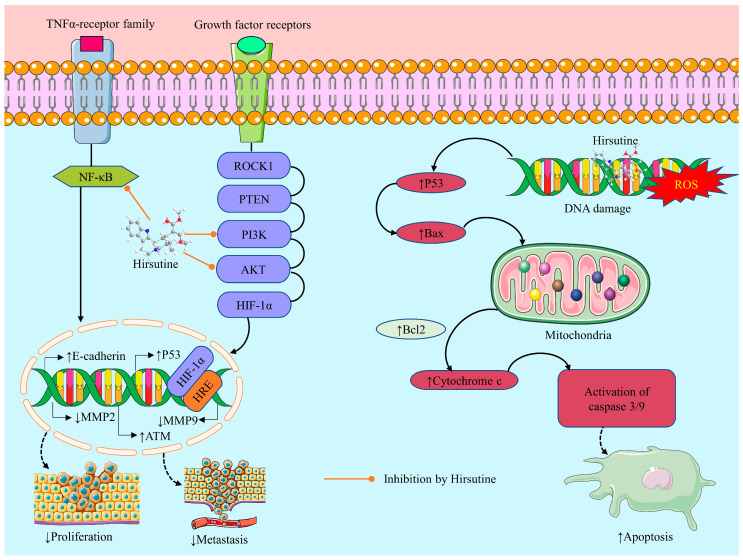
Possible anticancer mechanisms of hirsutine. ↓: Decrease/inhibition/downregulation; ↑: Increase/upregulation/stimulation; ROS: Reactive oxygen species; NF-κB: Nuclear Factor-kappa B; MMP2: Matrix Metalloproteinase 2; MMP9: Matrix Metalloproteinase 9; ATM: Ataxia Telangiectasia Mutated; HIF-1α: Hypoxia-Inducible Factor 1 alpha; AKT: Protein Kinase B; PTEN: Phosphatase and Tensin Homolog; PI3K: Phosphoinositide 3-Kinase; RE: Hypoxia-Response Element.

An investigation by Lou et al. (2014) demonstrated that HSN (25 µM) inhibits Mouse mammary carcinoma 4T1 cell migration in a dose-dependent manner. Furthermore, the results of the investigation showed that pre-treatment with HSN blocks 4T1 cell haptotaxis toward fibronectin in a Transwell chamber assay. Thus, the findings revealed that HSN pre-treatment significantly suppresses the migration and invasion activity of 4T1 cell lines by suppressing NF-κB signaling pathways which can be a treatment approach for breast cancer [20]. Similarly, a recent investigation by Huang et al. (2017) found that HSN with an IC_50_ value of 62.82 μM/L restricts hypoxia-mediated migration and invasion in human breast cancer MCF-7 cells, most likely through down-regulation of the protein levels of HIF-1α, MMP-9, and Snail and up-regulation of the protein level of E-cadherin [24]. Shown in Figure 3 are the possible anticancer mechanisms of HSN.

##### Anti-Proliferative Effect

By interfering with the normal cell cycle and causing cell death, chemotherapy medications target rapidly dividing cells, including cancer cells. Consequently, these drugs are often referred to as “anti-proliferative” medications, as they can inhibit or ease up the growth and proliferation of cancer cells [99]. In this respect, the cell counting kit-8 (CCK8) assay performed by Meng et al. (2021) demonstrated that HSN, at concentrations of 10, 25, and 50 μM, could remarkably suppress the proliferation of Jurkat clone E6-1 cells over 48 h. Similarly, flow cytometry experiments showed that HSN might cause apoptosis and G0/G1 phase arrest in Jurkat cells by lowering the Bcl-2 expression and, at the same time, enhancing Bax, mRNA, caspase-3, and -9 levels, thus inducing inhibition of cell proliferation in a tumor [25]. 

##### Genotoxic Effect

The ability of a chemical compound to damage the genetic material (DNA) of cells is known as genotoxicity. In this regard, numerous chemotherapy drugs may cause genotoxicity [100]. Genotoxicity can result in mutations, chromosomal aberrations, and DNA damage, which can promote cell death, cell transformation, and cancer [101]. Lou et al. (2015) found that HSN may induce genotoxicity. HSN at the concentration of 25, 12.5–50 μM damages the DNA of the HER2-positive/p53-mutated MDA-MB-453 cells by upregulating γH2AX expression and suppressing the NF-κB, HER2, and Akt pathways. HSN can also activate the p38 MAPK pathway in the MDA-MB-453 cells resulting in the induction of genotoxicity which destroys the DNA of breast cancer cells [22]. Another Investigation by Lou et al. (2016) stated that HSN (50 μM) induced the demise of MCF-7 cells and a sustained DNA damage response. This DNA damage was caused by interference with the ATM pathway and the production of ROS, which enhanced the anticancer effect of HSN in breast cancer cells [21].

#### 2.3.5. Effects on Thrombocytopenia

Thrombocytopenia is one of the most common hematological conditions, manifested by an unnaturally low platelet count due to several reasons [102]. Thrombocytopenia is linked to multiple syndromes and diseases and can be an early indicator of hematologic malignancies, thrombotic microangiopathies, infectious diseases, and autoimmune disorders, as well as a common adverse effect of numerous medications [103]. Megakaryocytes (MKs) are a type of functional hematopoietic stem cells; by differentiating and maturing MKs, it is possible to treat thrombocytopenia-related diseases [104]. In the hematopoietic system, transcription factors, cytokines, adhesion factors, and chemokines regulate MKs. The most essential of these mediators for the generation and differentiation of MKs is thrombopoietin, the ligand for the c-MPL receptor. Thrombopoietin is a key regulator of MKs differentiation and can trigger multiple signal transduction pathways, such as JAK2/STAT3/STAT5, MEK-ERK-FOG-1/TAL-1, and PI3K/AKT ((Table 3) [35,105,106]. MEK-ERK-FOG-1/TAL-1 signaling has been linked to MKs’ late differentiation and maturation [37]. A study by Kang et al. (2022) showed that HSN not only can promote thrombopoiesis by enhancing MKs differentiation and maturation of K562 and Meg01 cells through activation of MEK-ERK-FOG1/TAL1 signaling but can also lessen the decline of peripheral platelet concentration in mice. In addition, molecular docking simulations confirmed that HSN binds with high affinity to the signaling protein MAP kinase (MEK) [35]. Therefore, HSN can be a promising drug candidate for treating thrombocytopenia.

#### 2.3.6. Metabolic Disease and Disorders

##### Antihypertensive Effect

Hypertension is a severe health issue and a leading cause of premature death worldwide, with up to one-quarter of men and one-fifth of women, or approximately one billion people, suffering from the condition (https://www.who.int/health-topics/hypertension#tab=tab_1, accessed on 11 June 2023). Globally, hypertension is caused by a combination of factors, including long-term calorie consumption over energy expenditures, chronic supraphysiological ingestion of dietary salt, excessive alcohol use, and psychological stressors. Stroke, myocardial infarction, heart failure, renal insufficiency/failure, retinopathy, dementia, peripheral vascular disease, and premature death are only some of the many severe clinical outcomes associated with elevated BP, especially systolic BP [107]. When it comes to controlling heart activities, intracellular Ca^2+^ is pivotal. Vasoconstriction and an increase in vascular volume via the renin-angiotensin-aldosterone pathway contribute to a rise in vascular resistance and blood pressure as a result of an increase in Ca^2+^ influx into vascular smooth muscle cells [108,109]. Therefore, the regulation of Ca^2+^ influx is an important interventional approach to maintaining hypertension along with other cardiovascular diseases [110]. 

**Figure 4:**
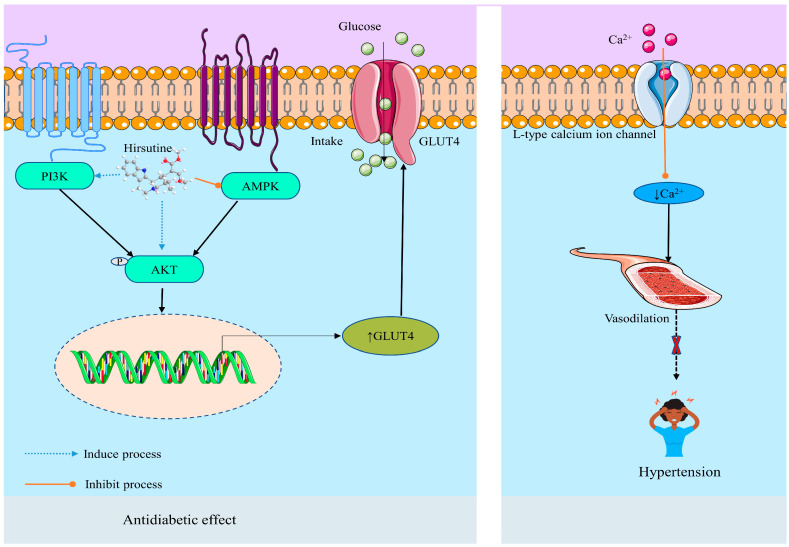
Possible antihypertensive and antidiabetic mechanisms of hirsutine. ↓: Decrease/inhibition/downregulation; ↑: Increase/upregulation/stimulation; AKT: Protein Kinase B; PI3K: Phosphoinositide 3-Kinase; GLUT4: Glucose Transporter 4.

Various studies have been reported about the antihypertensive effect of HSN, which is due to the inhibition abilities of L-type Ca^2+^ channels [27,32]. HSN (0.1 M to 10 M) altered the action potential waveform and lengthened the cycle of the rabbit SA node. Thus, for the first time, it was demonstrated that HSN exerts immediate inhibitory effects on the cardiac pacemaker. HSN reduced the levels of intracellular Ca^2+^ in isolated vascular smooth muscle cells by inhibiting Ca^2+^ influx via the L-type Ca^2+^ channel, producing a negative chronotropic effect [27]. In this context, Horie et al. (1992) reported that HSN (IC_50_ = 10.51.6 μM) inhibits the discharge of Ca^2+^ from the Ca^2+^ store and promotes Ca^2+^ uptake into the Ca^2+^ store in the smooth muscle of isolated rat aorta, resulting in a decrease of intracellular Ca^2+^ level by obstructing the voltage-dependent Ca^2+^ channel ((Table 3) [32]. A synthetic analog (referred to as compound 1 displayed in Figure 1) of HSN also displayed the antihypertensive effect by the same mechanism (blocking of Ca^2+^ influx through L-type Ca^2+^ channels) as HSN. The compound showed extraordinary activity on the contractile response of thoracic aorta rings from male SD rats in vitro reducing the systolic BP and heart rate [14]. Another study on the aortic arteries of rats by Yano et al. (1991) also demonstrated that HSN at a level of 10^−6^ to 3 × 10^−5^ M provided a vasodilation effect by inhibiting the trans-membrane Ca^2+^ influx via voltage-dependent Ca^2+^ channels [111]. The antihypertensive mechanism of HSN is depicted in Figure 4. 

##### Anti-Diabetic Effect

Type 2 diabetes mellitus (T2DM) is a metabolic illness with a worldwide incidence that is characterized by high blood sugar levels and insulin resistance (IR) in target tissues and is typically associated with a high risk of multiple complications [112,113,114]. It is necessary to investigate innovative diabetes treatments. In this respect, reducing hyperglycemia and controlling insulin resistance (IR) are two important steps in the treatment of diabetes [26,115]. An in vivo investigation conducted by Hu et al. (2022) revealed that administration of HSN (5, 10, and 20 mg/kg, p.o.) to HFD-induced diabetic rodents reduces body weight gain, hyperglycemia, and IR. In addition, the study indicated that HSN reverses IR-related hepatic steatosis and enhances *left ventricular* (LV) mass in liver and cardiac examinations [26]. An in vitro study by the same researchers showed that HSN at the concentration of 0.325 μM develops hepatic IR by activating the PI3K/Akt/GSK3β insulin signaling pathway, increasing glycogen synthesis, glucose consumption, and suppressing gluconeogenesis in insulin-resistant HepG2 cells. Similarly, in HGHI H9c2 cells, HSN activated both PI3K/Akt/GSK3β and AMPK/ACC signaling pathways to promote glucose uptake. Furthermore, enhancement of glycolysis was noted in both H9c2 and HGHI HepG2 cells treated by HSN (Table 3). These findings demonstrated the efficacy of HSN in alleviating hepatic and cardiac IR in vivo and in vitro, providing insights into the development of HSN as an IR prevention and treatment for diet-induced diabetes [26]. The anti-diabetic mechanism of HSN is displayed in Figure 4.

**Table 3:** Different pharmacological activities of hirsutine and their mechanisms.

Related Disease/Effect	Test Medium/Cell Line/Test System	Compound/ Dose (R/A)/IC_50_/ Concentration/ Course Interval	Possible Mechanism	Reference
Inflammation	Rats, LPS-induced preeclampsia	35, 70, and 140 mg/kg b.w.	↓ TNF-α, and ↓ IFN-γ, ↓ IL-6, ↓ IL-1β	[18]
	Rat brain microglia (LPS-induced inflammation, 10 µg/mL), in vitro	-	↓ NO, ↓ PGE2 and ↓ IL-1β, ↓ ROS, ↓ phosphorylation of the MAPK, ↓ Akt signaling proteins.	[29]
Thrombocytopenia	The Kunming thrombocytopenia mouse model was established by X-ray irradiation, in vivo	-	↑ MKD/MKM of K562 and Meg01 cells, ↑ platelet levels, ↑ MKD via activation of MEK-ERK-FOG1/TAL1 signaling	[35]
Neuronal death	Rat cerebellar granule cells (glutamate-induced neuronal death), in vitro	10^−4^–3 × 10^−4^ M	↓ Ca^2+^ influx	[36]
Myocardial ischemia-reperfusion	Sprague Dawley Rat Model	5, 10, and 20 mg/kg (p.o.)	↓ Myocardial infarct size, ↑ cardiac function, ↓ LDH, ↓ ROS, ↓ apoptosis, ↑ myocardial ATP, ↑ Mfn2 expression, ↓ p-Drp1, ↑ p-CaMKII, ↓ AKT/ASK-1/p38 MAPK pathway	[28]
Cardiomyocytes cell death	Neonatal rat cardiomyocytes treated with hypoxia	0.1, 1, and 10 μΜ	↓ Bax, ↓ Fas, ↓ caspase-3. ↑ Bcl-2.	[31]
Hypertension/ negative chronotropic/ antiarrhythmia	In male SD rats, in vitro, vasodilatation induced by the NO/cyclic GMP pathway	IC_50_ = 1.129×10^−9^ ± 0.5025	↓ Ca^2+^ influx, no effect on K^+^ channel	[14]
	Male Japanese white rabbits	0.1 to 10 μM	↓ Influx of Ca^2+^ via voltage-dependent Ca^2+^ channels	[27]
	Male Wistar rats	30 μM	↓ Intracellular Ca^2+^ influx	[32]
	Aortic arteries of Wistar male rats, in vitro	10^−6^ to 3 × 10^−5^ M	↓ Ca^2+^ influx	[112]
	Male Sprague-Dawley rats	3–300 µM, y 60 mM KCl (IC_50_ = 20–30 µM)	↑ Ca^2+^, ↑ KCl	[33]
Diabetes	Male C57BL/6 J mice, high-fat diet-induced diabetes, in vivo, *n* = 9	5, 10, and 20 mg/kg (p.o)	↓ Ca^2+^, ↓ glucose tolerance, ↑ glucose uptake, ↑ glycolysis,↑ phosphatidylinositol 3-kinase (PI3K)/Akt pathways	[26]
	HepG2 and H9c2 cells, high-glucose and high-insulin (HGHI) incubation, in vitro	0.325 μM	↑ p-Akt, ↑ GLUT4 activity, ↓ AMPK.	
Antiviral activity	Human lung carcinoma cells (A549) and baby hamster kidney cells (BHK-21), DENV-1 (02-20 strain), DENV-2 (16681 strain), DENV-3 (09-59 strain), and DENV-4 (09-48 strain)	10 µM	↓ Ca^2+^, ↓ viral particle assembly, ↓ budding, or release step.	[19]
	Influenza A virus (subtype H3N2), in vitro	EC_50_ = 0.4–0.57 µg/mL	↓ Replication of the strains of Influenza A	[82]
Antitumor	Jurkat clone E6-1 cells, evaluated by CCK8 assay, in vitro	10, 25, and 50 μM for 48 h	↓ Cell proliferation, ↑ pro-apoptotic Bax, cleaved-caspase3, cleaved-caspase9 and Cyt C proteins, ↓ Bcl-2	[25]
Lung cancer	A549 xenograft mouse model, NCI-H1299, and LO2 cells	60–80 μM	↑ Apoptosis, ↑ ROCK1 and PTEN,↓ PI3K/Akt, ↑ caspase-3	[34]
Breast cancer	MCF-10A, MCF-7 and MDA-MB-231 cells	160 μM/L HSN for 24, 48, and 72 h	↑ Apoptosis, ↓ Bax, ↓ Bcl-2, opening MPTP, releasing Cyt C from mitochondria, and activating caspase 9 and caspase 3.	[23]
	MCF-7	IC_50_ = 62.82 μM/L	Inhibits hypoxia, ↓ migration, and ↓ invasion, ↓ HIF-1α, ↓ snail, ↓ MMP-9, ↑ E-cadherin	[24]
	MCF-7 cell line, ataxia telangiectasia mutated (ATM) pathway	(50 µM) for 24 h	↑ Cell apoptosis by inducing DNA damage, ↑ ATM pathway, ↑ p53-independent DNA damage response, ↑ ROS, ↓ metastasis of breast cancer cells	[21]
	HER2-positive/p53-mutated MDA-MB-453 and BT474 cell lines, in vitro	6.25, 12.5, 25, and 50 μM	↑ Cytotoxicity, ↑ apoptosis, ↑ DNA damage response	[22]
	Mouse mammary carcinoma 4T1 cells, in vitro	(25 µM) for 24 h	↓ NF-κB, ↓ migration and invasion. ↓ MMP-2, MMP-9, and ↓ NF-κB signaling pathways	[20]

LPS: Lipopolysaccharide; ROS: Reactive Oxygen Species; NO: Nitric Oxide; PGE2: Prostaglandin E2; Akt: Ak strain transforming; IL-1β: Interleukin 1β; I_k_B: Inhibitor of Nuclear Factor-κB; NF-κB: Nuclear factor kappa B; DENV: Dengue Virus; BHK: Baby Hamster Kidney Cells; HER2: Human Epidermal Growth Factor Receptor 2; BBB: Blood-Brain Barrier; TNF-α: Tumor Necrosis Factor α; IFN-γ: Interferon γ; ATM: Ataxia Telangiectasia Mutated; MMP-2: Matrix metalloproteinase-2; ROCK: Rho-Associated Protein Kinase; PTEN: Phosphatase and Tensin Homolog; Bax: Bcl-2-associated X protein; Bcl-2: B-cell lymphoma 2; PI3K: Phosphoinositide 3-Kinases; MKM: Microdosimetric Kinetic Model; MKD: Mevalonate Kinase Deficiency; NGF: Nerve Growth Factor; CCK: Cell Counting Kit; DRG: Dorsal Root Ganglion; P-gp: P-Glycoprotein; STZ: Streptozotocin; LDH: Lactate Dehydrogenase; Mfn2: Mitofusin2; p-Drp1: Dynamin-Related Protein 1 Phosphorylation; p-CaMKII: Protein Kinase II Phosphorylation; HepG2: The human hepatocellular carcinoma cell line; ADR: Adriamycin-Resistant; MDR: Multidrug Resistance; HFD: High-Fat Diet; HGHI: High-Glucose and High-Insulin; AMPK: AMP-Activated Protein Kinase; MPTP: Mitochondrial Permeability Transition Pore; MAPK: Mitogen-activated protein kinases.

## 3. Toxicological Profile 

Toxicity testing is a crucial component in the identification of potential adverse reactions induced by chemical substances. For example, the manifestation of carcinogenicity, genotoxicity, immunotoxicity, and reproductive and developmental toxicity in humans is commonly found following prolonged exposure to chemicals [116]. The principal aim of toxicology investigations in the drug development process is to assess the safety profile of potential drug candidates [117]. This is achieved using animal models and validated methods [118]. Alkaloids are one of the greatest classes of secondary metabolites found in plants and are present in several economically significant plant families. Due to their toxicity, alkaloids can serve as defense compounds in plants, being effective against pathogens and predators [119]. The observed toxicity of alkaloids has been documented in both animals and humans [120]. The toxicological effects of alkaloids are contingent upon various factors, including the precise quantity administered, duration of exposure, and individual attributes such as sensitivity, location of action, and developmental stage [119].

Research findings showed that HSN exhibits cytotoxicity against different cell lines (NCI-H1299, MCF-10A, MCF-7, MDA-MB-231, and 4T1) at doses ranging from 6.25 to 80 μM in a number of in vitro studies [22,23,34]. However, HSN in vivo was not toxic for normal tissues in lower doses [121]. Acute toxicity analysis of HSN in a mouse model indicated that the LD_50_ of HSN is 110 mg/kg (i.p.) and 35 mg/kg (i.v.) (https://pubchem.ncbi.nlm.nih.gov/compound/3037884#section=Acute-Effects, accessed on 10 August 2023). On the other hand, Compound **1** exerted no toxicity on cell viability in the MTT assay at the normal dosage range. In an in vitro experiment, when VSMCs were treated with compound **1** (Figure 1) (1.6, 8, 40, 200, 1000 μΜ) for 12 h, only the 1000 μΜ group showed toxicity on the cells compared with the control group [14]. This indicates that compound **1** will be an ideal and safe medicine candidate.

## 4. Methodology

### 4.1. Literature Searching Strategy

The literature search in known databases such as PubMed, Springer Link, Scopus, Wiley Online, Nature, Web of Science, ScienceDirect, and Google Scholar was accomplished using the keyword “hirsutine”, then paired with “anti-inflammatory activity”, “antioxidant”, “oxidative stress”, “protective effect”, “gastritis”, “tumor”, “gastroprotective activity”, “renoprotective activity”, “hepatoprotective activity”, “cardioprotective effect”, “hepatoprotective activity”, “antimicrobial effect”, “osteoprotective activity”, “antiviral effect”, “biological activities”, “pharmacological effects”, “pharmacological activities”, “biological sources”, “neurological effect”, “pulmoprotective effect”, “antidiabetic effect”, cerebral ischemia/reperfusion”, “chemical features”, “pharmacokinetics”, “in vivo studies” or “in vitro studies”. In our search, no language limitations were imposed. The studies have been assessed in depth, with information regarding the sources, dose, concentration, test system, the proposed mechanism of pharmacological activities, and overall conclusion.

### 4.2. Inclusion and Exclusion Criteria

Inclusion criteria involved: (1) Studies performed in vitro, ex vivo, or in vivo and in silico with or without employing laboratory animals, including mice, rats, rabbits, and humans, and their derived tissues or cells, (2) Studies with pharmacological activities and botanical sources of HSN, (3) Studies with HSN or its derivatives or preparations, (4) Studies with HSN isolated from natural sources, (5) Studies showing the presence of HSN as a bioactive compound in preclinical studies of plant extracts, (6) Studies indicating that HSN or its derivatives exhibit synergistic effects when combined with other chemical compounds, (7) Studies with or without hypothesized mechanisms of action, (8) Studies carried out on the botanical sources of HSN, and (9) Studies related to the pharmacokinetics of HSN. On the other hand, exclusion criteria included (a) Duplicated data, titles, and/or abstracts which did not meet the inclusion criteria, (b) Papers written in languages other than English, (c) Case reports, letters, editorials, and commentaries, and (d) Studies without full text available. 

### 4.3. In Silico ADME Prediction

In silico ADME of HSN is also predicted through the SwissADME online tool (http://www.swissadme.ch/index.php, accessed on 12 June 2023) to evaluate the PK properties of HSN [122].

### 4.4. Database Reports

**Figure 5:**
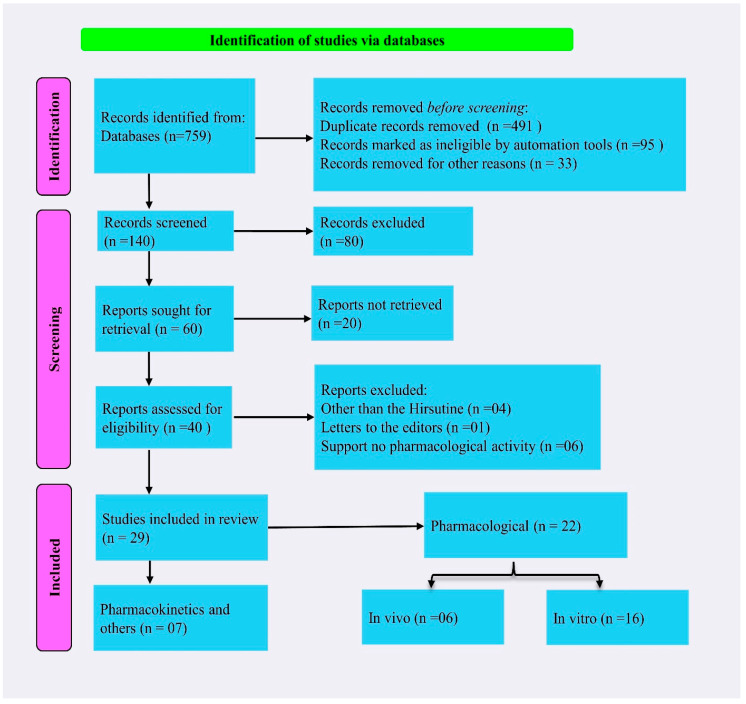
PRISMA analysis of the collected data of hirsutine.

A total of 759 scientific articles were collected as of 1 July 2023, from databases. After that, 96.18% of collected articles were eliminated due to duplication of reports, irrelevant information, lack of sufficient information, and automation systems deeming them unsuitable. Based on the inclusion criteria, we included information in this study from a total of 29 articles on HSN. Among the included articles, 75.86% reported the pharmacological activities of the compound, and 24.14% reported pharmacokinetics (PK), biological sources, and others. In the pharmacological investigation, 27.28% was done in vivo and 72.72% in in vitro test systems. The Preferred Reporting Items for Systematic Reviews and *Meta*-*Analyses* (PRISMA) analysis of the collected data of HSN is displayed in Figure 5.

## 5. Conclusions 

At present time, many people depend on intrinsic foodstuffs in the fight against diseases. Thus, people are encouraged to consume natural food products obtained from fruits and vegetables and other food items to treat and manage cardiovascular disorders, cancer, and immune dysfunction, among others. These natural compounds are safer and less expensive than synthetic drugs. The purpose of the present study was to evaluate the therapeutic potential of HSN by investigating the available data from various preclinical studies and the underlying mechanisms behind these effects. Results demonstrated that HSN exhibits various pharmacological activities, including antioxidant, anti-inflammatory, antiviral (against various serotypes of dengue and influenza A viruses), antidiabetic, anticancer, cardioprotective, and stimulating thrombopoiesis via activation of MEK-ERK-FOG1/TAL1 signaling. In addition, it exerts potent activity in preventing various neurogenerative diseases, especially ischemia/reperfusion, neuroinflammation, and neurotoxicity. Furthermore, findings from this study showed that HSN is a potent blocker of Ca^2+^ influx via the L-type Ca^2+^ channels, resulting in reduced hypertension, neuronal cell death, and AMI. In terms of anticancer effects, HSN exhibits potency against breast cancer mainly through the induction of oxidative stress (when it is used in elevated concentration), inhibition of migration and invasion through cytotoxic, apoptotic, genotoxic, and antiproliferative effects. Pharmacokinetics studies demonstrated that HSN is well absorbed and distributed in various organs of the body, but the oral bioavailability of the compound was reported to be low due to the metabolism of the compound in the liver as well as the fact that the compound can cross the BBB. Therefore, the development of an alternative route is needed to increase the bioavailability and efficacy of the lead. However, extensive clinical studies are necessary to establish its efficacy for long-term use in treating human diseases.

## Figures and Tables

**Table 1:** Various botanical sources of hirsutine.

Plants	Parts	References
*Uncaria rhynchophylla* (Miquel)	Bark	[25]
	Dried hooks	[32]
*U. hirsuta* *U. lancifolia*, *U. scandens*, *U. macrophylla* *U. homomalla*, *U. laevigata*, *U. sessilifructus*, *U. yunnanensis* *U. lanosa*, *U. rhynchophylloides*,	Stems and hooks	[44]
*U. sinensis*	Stems and hooks	[36]
*Uncaria tomentosa*	Leaves and roots	[45]
*Mitragyna hirsuta*	Leaves and root	https://pubchem.ncbi.nlm.nih.gov/taxonomy/371154, accessed on 30 April 2023

**Table 2:** Different parameters of HSN and their values/status of ADME predicted by SwissADME.

Parameter (s)	Values/Status
**Physicochemical properties**	
Molecular mass	368.5 g/mol
Number of heavy atoms	27
Number of aromatic heavy atoms	9
Number of rotatable bonds	5
Number H-bond acceptors	4
Number H-bond donors	1
Molar Refractivity	110.39
TPSA	54.56 Å^2^
**Lipophilicity**	
Log Po/w (MLOGP)	2.35
**Water Solubility**	
Solubility class	Moderately soluble
**Pharmacokinetics**	
GI absorption	High
BBB permeant	Yes
P-gp substrate	Yes
CYP1A2 inhibitor	No
CYP2C19 inhibitor	No
**Drug-likeness**	
Lipinski	Yes; 0 violation
Bioavailability Score	0.55

## Data Availability

The processed data are available from the corresponding author upon request.
